# Custom-made health-care: an experimental investigation

**DOI:** 10.1186/s13561-020-00299-4

**Published:** 2020-12-18

**Authors:** Claudia Keser, Claude Montmarquette, Martin Schmidt, Cornelius Schnitzler

**Affiliations:** 1 Department of Economics, Universität Göttingen, Platz der Göttinger Sieben 3, D-37073 Göttingen, Germany; 2 CIRANO, 1130, Sherbrooke West, office 1400, Montréal, H3A 2M8 Canada; 3 University of Montreal, Montreal, Canada; 4 Present address: KIT, Fritz-Erler-Str. 1-3, D-76133 Karlsruhe, Germany; 5 Present address: Arkansas Economic Development Commission, Unter den Linden 10, D-10117 Berlin, Germany

**Keywords:** Experimental economics, Physician reimbursement, Capitation, Fee-for-service, Fee regulation

## Abstract

**Background:**

Physicians’ financial interests might conflict with the best service to patients. It is essential to gain a thorough understanding of the effect of remuneration systems on physician behaviour.

**Methods:**

We conducted a controlled laboratory experiment using a within-subject design to investigate physician behaviour underpayment heterogeneity. Each physician provided medical care to patients whose treatments were paid for under fee-for-service (FFS) or capitation (CAP).

**Results:**

We observed that physicians customized their care in response to the payment system. FFS patients received considerably more medical care than did CAP patients with the same illness and treatment preference. Physicians over-served FFS patients and under-served CAP patients. After a CAP payment reduction, we observed neither a quantity reduction under CAP nor a spillover in FFS patients’ treatment.

**Conclusions:**

The results suggest that, in our experimental model, fee regulation can be used to some extent to control physician spending since we did not identify a behavioural response to the CAP payment cut. Physicians did not recoup lost income by altering treatment behaviour toward CAP and/or FFS patients. Experimental economics is an excellent tool for ensuring the welfare of all those involved in the health system. Further research should investigate payment incentives as a means of developing health care teams that are more efficient.

**Supplementary Information:**

The online version contains supplementary material available at 10.1186/s13561-020-00299-4.

## Background

Health care spending constitutes a significant share of GDP. In 2018, it amounted to about 17% in the USA, 11% in Germany, and 9% in OECD countries [1]. A large share of this money goes to the payment of health care providers. Since physicians’ financial interests might conflict with the best service to patients [2], it is essential to understand the effect of financial incentives on physician behaviour.

There is extensive literature on this issue. In a theoretical model, Ellis & McGuire [3] demonstrated that FFS leads to an over-provision of medical services, whereas CAP results in an under-provision. Numerous empirical studies have shown that physicians tend to respond to financial incentives in this way [4–8].

In an early controlled laboratory experiment on financial incentives to health care providers, Fan et al. [9] investigated physicians’ provision of virtual medical services under two alternative methods for controlling the cost of physician services under global budgeting. They found that subjects provided more medical services under the expenditure-cap policy than under the expenditure-target method. In a seminal paper, Hennig-Schmidt, Selten & Wiesen ([10], HSW hereafter) studied the effect of the two alternative remuneration systems, fee-for-service (FFS) and capitation (CAP), on the quantity of medical services provided by physicians. FFS is a volume-based physician payment system in which physicians are paid separately for each unit of medical service rendered. Under CAP, physicians receive a lump-sum payment for a patient’s treatment, irrespective of the quantity of services rendered. In the experiment, medical students decided on the quantity of medical services to be provided to each virtual patient on a given patient list. HSW (2011) shows that physicians paid via FFS provided more medical services than those paid via CAP. Furthermore, FFS physicians tended to over-serve patients, while capitated physicians had a propensity to under-serve patients.

In our paper, we present an experiment that builds on the study by HSW (2011) by extending it in two directions, payment heterogeneity and fee regulation (i.e., a cut in the lump-sum payment under CAP). HSW (2011) examined treatment decisions in a setting where physicians faced a single payment system, either FFS or CAP. Since physicians in real life frequently face multiple payment systems, we tested the robustness of the findings in HSW (2011) by creating an environment with payment heterogeneity. In our experiment, each physician faced patients under both payment systems, FFS and CAP. In this heterogeneous payment setting, we wanted to investigate whether physicians customize care in response to financial incentives at the individual patient level or provide ready-to-wear treatment [11], that is, a one-for-all approach rather than selecting treatment on a patient-by-patient basis.

Our experiment’s heterogeneous setting also made studying the effect of fee regulation more interesting than it would be in a homogeneous setting. At one point in the experiment, we announced a reduction in the lump-sum payment for all patients under CAP to explore whether and how the physicians would react to such a reduction. We were not only interested in whether physicians tried to offset the lost income by reducing the provision of care to CAP patients, but also whether the lump-sum payment reduction had any (spillover) effect on their provision behaviour under FFS.

In the experiment, each FFS patient received considerably more medical care than did the corresponding CAP patient with the same illness and treatment preference. On average, FFS patients received more than twice as many medical services as CAP patients. In general, physicians over-served FFS patients and under-served CAP patients. However, we did not identify any behavioural response to the CAP payment cut. We detected no quantity offset under CAP nor spillover under FFS. This suggests that, in our experimental model, fee regulation can be used to some extent as a means of controlling spending on physician services without reducing the overall quantity of care. This element brings added value to all recent experimental studies using the basic experimental platform developed by HSW (2011). In addition to the typical concerns regarding doctors and patients’ benefits, it involves something of use to the taxpayers who pay for the public health system. We are aware of one recent study by Brosig-Koch et al. [12] that introduces a variety of bonus pay amounts to a CAP payment system. The results of this study suggest that, in keeping with Keser et al. [13], performance pay may enhance the quality of care. In addition, they suggest that the size of the bonus does not matter, which is in keeping with our finding. Interestingly, under the FFS payment system, Di Guida et al. [14] observe that fee size has an effect on over-provision.

Following Ellis & McGuire [3], the theoretical modelling of physicians’ behaviour should be based on the assumption that they take both their financial interests and their patients’ interests into account. Therefore, to acquire some understanding of individual decision heuristics, we provide a classification of the physicians in our experiment based on their tendencies to maximize patient benefit or maximize their profit under each of the two payment systems. About half of the physicians are classified as profit maximizers, while most remaining physicians exhibit mixed motives. Benefit maximizers exist but are rare. This result relates to Godager & Wiesen [15], who find, based on the data of HSW (2011), “substantial variation in the degree of physician altruism”. Also, Martinsson & Persson [16] observe heterogeneity in altruism across physicians in their experiment; also, physicians’ degree of altruism varies across patients with different medical needs. Reif et al. [17] added to their experiment a third party representing the health insurance that finances medical service provision and find that physicians also consider the payoffs to the third party.

We are aware that the empirical literature on physician behaviour has reported conduct such as self-selection, cream-skimming, and adjustments to practice size in response to payment systems. However, our study focussed exclusively on the quantity of medical services provided. We used a standardized list of patients in our experiment to guarantee the comparability of the volume of services provided to similar patients under the two payment systems, before and after the CAP payment reduction.

## Methods

### Experimental model

Participants provided medical care to 36 sequentially presented virtual patients in a heterogeneous payment environment. The patients were presented in two sequences of 18 patients each, S1 and S2. In each of the two sequences, physicians faced patients whose treatments were paid for under either FFS or CAP. Under FFS, participants were paid separately for each unit of medical services provided. Under CAP, participants received a lump-sum payment, irrespective of the number of services provided. The payment system varied on a patient-by-patient basis. The two sequences, S1 and S2, comprised similar patients and differed exclusively in the lump-sum payment amount under CAP.

Each participant decided on the quantity q ϵ {0, 1, …, 10} of medical services to be provided to each patient. Treatment choices impacted both physician profit and patient benefit. Remuneration, profit, and patient benefit were measured in experimental currency units (ECUs).

The virtual patients were characterized by three attributes: payment system, illness, and treatment preference. The first attribute, the payment system, was either FFS or CAP. The second patient attribute was an illness. Patients suffered from one of three potential illnesses: A, B, or C. Illness impacted the FFS fee function, and thus the physician’s profit under FFS. We used three of the FFS fee functions examined by HSW (2011), which were chosen using reference points from the German scale of charges and fees for physician services. In contrast to Brosig-Koch et al. [18], there was no attempt to keep profit level and marginal profit under FFS and CAP directly comparable. In our experimental protocol, such control would not have been sustainable with the reduction in CAP payment.

We used the same FFS fee functions to characterize the patients in S1 and S2. For each illness, remuneration under FFS increased along with the number of medical services provided (see Table 1). Illness did not have an impact on remuneration under CAP. The lump-sum payment under CAP was independent of both illness and the number of services provided. However, it differed across S1 and S2, decreasing from 12.00 ECU in S1 to 9.60 ECU in S2 (20% decrease).

**Table 1:** Physician remuneration (in ECU)

			Quantity of medical services										
Rem. syst.	Seq.	Illness	0	1	2	3	4	5	6	7	8	9	10
FFS	S1,S2	A	0.00	1.70	3.40	5.10	5.80	10.50	11.00	12.10	13.50	14.90	16.60
FFS	S1,S2	B	0.00	1.80	3.60	5.40	7.20	9.00	10.80	12.60	14.40	16.20	18.30
FFS	S1,S2	C	0.00	2.00	4.00	6.00	8.00	8.20	15.00	16.90	18.90	21.30	23.60
CAP	S1	A,B,C	12.00	12.00	12.00	12.00	12.00	12.00	12.00	12.00	12.00	12.00	12.00
CAP	S2	A,B,C	9.60	9.60	9.60	9.60	9.60	9.60	9.60	9.60	9.60	9.60	9.60

The third patient attribute was treatment preference. We distinguished between three patient types, each characterized by a particular treatment preference, with different benefit functions (B_1_(q), B_2_(q), B_3_(q)). These were adopted from HSW (2011). The patient benefit function B_i_(q) describes the benefit that a patient of type i (i ϵ {1, 2, 3}) draws from treatment quantity q and was measured in ECU. The different benefit functions imply that patients, independent of illness, respond differently to treatment quantity. The same benefit functions were used to characterize the patients in S1 and S2.

Each of the three benefit functions was designed to have a global optimum q_i_*, which determined the treatment preference, i.e., the “right” amount of medical care for each patient type. Specifying global optima in the interior of the action space allowed us to observe any potential over- and under-provision of medical care for each patient type.

**Fig. 1:**
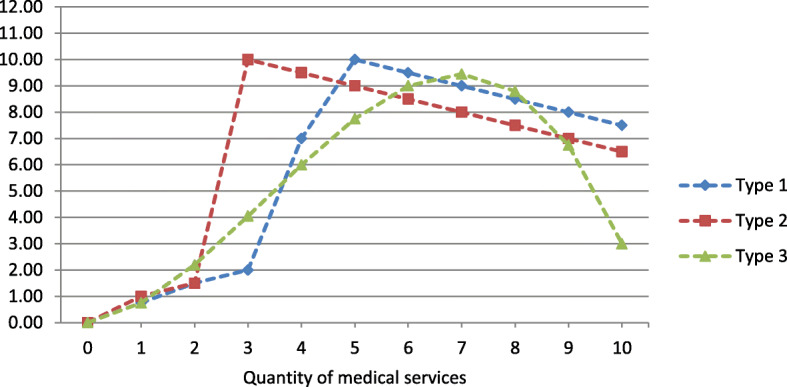
Patient benefit functions in ECU

The benefit functions for type 1 and type 2 patients were designed such that the monetary benefit drop-off from the optimal level was smaller in the case of over-provision than in the case of under-provision. The reverse was true for the monetary benefit of type 3 patients (see Fig. 1 or Additional file 1: Table B1).

The patients in our experiment were characterized by two payment systems, three illnesses, and three types of treatment preferences. Each of the 2 × 3 × 3 combinations of payment system, illness, and preference represented an individual patient in each of the two sequences. Participants thus faced a heterogeneous patient population with 18 individual patients showing different characteristics in each of the two sequences. Under this design, half of the patients in a sequence were treated under FFS and the other half under CAP.

The patients were passive and fully insured, accepting any quantity of medical services provided. Physicians’ treatment choices impacted both patient benefit and physician profit. Physicians were confronted with a convex cost function, c (q_j_) = 0.1 q_j_^2^, where q_j_ is the amount of medical services provided to patient j. This function was, again, adopted from HSW (2011). The cost function remained unaffected by the payment system, illness, patient type, and sequence, implying that it was the same for treating all patients in the experiment.

**Fig. 2:**
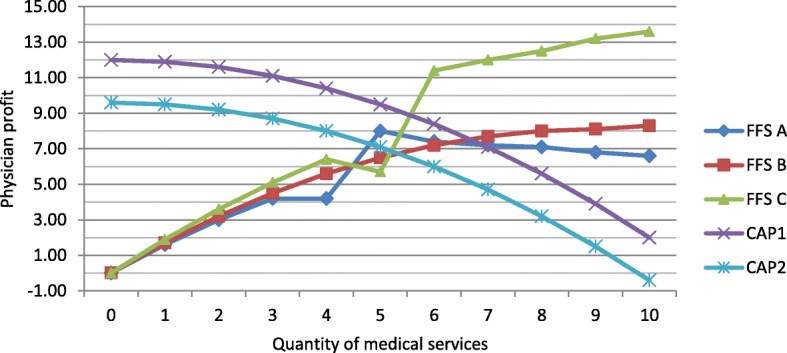
Physician profit functions

Physician profit under FFS varied across illnesses and was identical in both sequences. Profit under CAP remained unaffected by illness but varied across sequences (see Fig. 2 or Additional file 1: Table B2). The FFS functions were designed such that the profit function displayed a trade-off between maximum patient benefit and maximum physician profit in all cases but one (FFS patient type 1 with illness A). Under CAP, physicians had to deviate from the profit-maximizing quantity to create patient benefit.

### Experimental procedure

We conducted the experiment at the CIRANO lab in Montreal, Canada, using the z-Tree software program [19]. Participants were 23 students with an academic background in a health care discipline.

Before the experiment, the procedure was as follows: Participants and the experimenter gathered in a conference room where the instructions (see online Additional file 1) were distributed and read out to participants. From this moment on, participants were not allowed to communicate with each other and were instructed to refrain from publicly raising questions regarding the instructions. Each participant was randomly assigned to one of the isolated workstations in the laboratory. The layout of the workstations made visual contact and communication between participants impossible.

After reading the instructions, participants were presented with a programmed questionnaire regarding the instructions. The experimenter was available to resolve any open questions regarding the instructions individually. The experiment began once all participants correctly answered all the questions on the questionnaire.

Participants were informed in the instructions that the aggregate patient benefit would be donated to a charitable health care organization. This was designed to encourage them to take patient benefits into account. At the beginning of the experiment, each participant selected a charitable health care organization (Canadian Cancer Society, Multiple Sclerosis Society of Canada, or Parkinson Society of Canada). After the experiment, each participant received an electronic transfer confirmation of the donation to the individually chosen organization.

In the experiment, participants allocated medical services to 36 virtual patients in two consecutive sequences. Patients were presented one after the other in each sequence. The patients’ order was randomized in the first sequence (S1) and repeated in the second sequence (S2). The relevant payment system was revealed for each patient. Neither illness nor patient type, however, was specified in detail. Each virtual patient was presented as a table that listed the physician profit and patient benefit associated with the patient’s characteristics for each possible quantity of medical services. Participants decided on the quantity of medical services based on these numbers. Between the first and second sequence, participants were notified of a lump-sum payment reduction under CAP. Participants carried on with the experiment after acknowledging the payment cut.

For each participant, physician profit and patient benefit were tallied up separately and converted into CAN$, applying a conversion factor of CAN$0.02 per ECU. As communicated in the instructions, each participant privately received, in cash, a payment from the experiment in addition to a CAN$5.00 show-up fee at the end of the experiment. The patient benefits generated by participants who had selected the same charitable health care organization were pooled and donated to the respective organization.

We conducted two experimental sessions, with 10 and 13 participants, respectively. Each session lasted approximately 75 min. Participants earned, on average, CAN$18.27, including the show-up fee. The donations (of the aggregate patient benefit) to the charitable health care organizations totaled CAN$239.47.

### Hypotheses

An individual who disregarded the effect of their treatment decisions on patient benefit would maximize profit by generating zero ECUs, irrespective of the amount of the lump-sum payment, under capitation, and 5 or 10 ECUs, depending on the patient’s illness, under FFS. However, the findings of HSW (2011) show that experimental physicians do take patient benefit into account, which we should also expect from real-life physicians due to professional ethics. Consequently, we anticipate that our experiment subjects will choose to provide more than zero units of medical care to CAP patients and deviate from the profit-maximizing quantities for FFS patients.

In keeping with HSW (2011), Hennig-Schmidt & Wiesen [20], Brosig-Koch et al. [18, 21] and Wang et al. [22], as well as the theoretical predictions of Ellis & McGuire [3], we expect experimental physicians to over-serve FFS patients and under-serve CAP patients, thus providing more services to FFS patients than similar CAP patients in our experiment.

#### Hypothesis 1

In a heterogeneous payment environment, physicians over-serve patients whose treatments are paid for under FFS, and under-serve those whose treatments are paid for under CAP.

Frank & Zeckhauser [11] empirically showed that, in the case of depression, some physicians engage in highly suboptimal therapeutic practices (a one-for-all approach), while others customize care, depending on the costs of doing so. Hypothesis 1 directly implies Hypothesis 2.

#### Hypothesis 2

Physicians do customize care in response to the payment system in a heterogeneous payment environment: in each sequence, physicians provide more medical services to FFS than to CAP patients.

The theoretical analysis by Ellis & McGuire [3] was based on the assumption that the utility function of a physician includes both the patient’s benefit and the physician’s profit. Given this assumption, we expect, for the lump-sum payment reduction under CAP, a reduction in the quantities of services provided to CAP patients. We also anticipate an impact on FFS patients’ treatment decisions since physicians could use the “FFS market” as an avenue to make up for lost CAP income [23–25]. Hypotheses 3 and 4 follow.

#### Hypothesis 3

Physicians’ provision behaviour is affected by an ex-ante payment reduction under capitation. Physicians do alter their treatment behaviour toward FFS and CAP patients in order to recoup lost income. We expect a decline in CAP patients’ treatment quantities and a spillover effect in the FFS payment system.

#### Hypothesis 4

On an individual level, some physicians will be profit-oriented, while others will be patient benefit-oriented.

### Classification of individual physician behaviours

To examine whether individual physicians showed any tendencies to maximize patient benefit or physician profit, we constructed two qualitative evaluation criteria. The first criterion looks at whether a physician tends to maximize patient benefit. This criterion applies to the treatment of both CAP and FFS patients. We calculated the average number of services provided to patients of the same patient type, payment system, and sequence for each physician. Each combination of payment system and sequence yielded three measures, one for each patient type. Using these measures, we concluded that a physician was “benefit-oriented” (under a payment system in a given sequence) if the average number of medical services increased along with the patient type’s need for medical attention.

The second criterion identifies a potential tendency to maximize physician profit. This criterion applies to the treatment of FFS patients. We compared, for each physician, the average number of treatments provided to FFS patients in a sequence suffering from illness A, with the average quantities provided to FFS patients suffering from illness B or C. We concluded that a physician was “profit-oriented” if the average quantity provided to patients suffering from illness B or C (who should be maximally treated to maximize the physician’s profit) exceeded the average number of services provided to patients suffering from illness A (who should be treated with 5 units to maximize the physician’s profit).

Table 2 shows, for each physician, the observed tendencies (benefit-oriented under CAP and FFS, and profit-oriented under FFS) in each of the two sequences. A checkmark indicates that the individual physician displayed the orientation in question, while an X implies that the individual physician did not exhibit it.

**Table 2:** Behavioural tendencies

Physician #	*Patient benefit-oriented*		*Profit oriented*	*Patient benefit-oriented*		*Profit-oriented*
	CAP@1	FFS@1	FFS@1	CAP@2	FFS@2	FFS@2
1	✓	**X**	✓	✓	**X**	✓
2	**X**	**X**	✓	**X**	**X**	✓
3	**X**	✓	**X**	**X**	**X**	**X**
4	**X**	**X**	✓	**X**	**X**	✓
5	✓	**X**	✓	**X**	**X**	✓
6	**X**	**X**	✓	**X**	**X**	✓
7	**X**	**X**	✓	**X**	**X**	✓
8	**X**	**X**	✓	**X**	**X**	✓
9	**X**	✓	✓	**X**	✓	✓
10	✓	✓	**X**	✓	✓	✓
11	✓	✓	**X**	✓	✓	**X**
12	✓	✓	✓	✓	✓	✓
13	✓	**X**	✓	**X**	**X**	✓
14	✓	**X**	✓	✓	✓	✓
15	**X**	**X**	✓	**X**	**X**	✓
16	**X**	**X**	✓	**X**	**X**	✓
17	✓	✓	✓	**X**	**X**	✓
18	**X**	**X**	✓	**X**	**X**	✓
19	✓	**X**	✓	**X**	**X**	✓
20	**X**	**X**	✓	**X**	**X**	✓
21	**X**	**X**	✓	**X**	**X**	✓
22	**X**	**X**	✓	✓	**X**	✓
23	✓	✓	✓	✓	✓	✓

The physicians are classified into three broad categories: benefit maximizers, profit maximizers and (regular) mixed-motive deciders. We identified a physician as a benefit maximizer if the physician (in a given sequence) was benefit-oriented under both payment systems and not profit-oriented. In S1 (S2), we found two (one) benefit maximizers.

We identified a physician as a profit maximizer if the physician’s provision behaviour was profit-oriented without maximizing benefit. We found 11 (14) profit maximizers in S1 (S2). Note that these profit maximizers, except for two (physicians #13 and #19), generated on average at least 90% of the optimal profit over the two sequences.

We identified a physician as a (regular) mixed-motive decider if the physician’s observed treatment behaviour was benefit-oriented under CAP, exclusively, and profit-oriented under FFS. We observe five (two) mixed-motive deciders in S1 (S2).

A small number of physicians did not fall into any of the three categories. Three (four) physicians were benefit-oriented for both CAP and FFS and profit-oriented at the same time. In addition, a single physician (#9) was benefit-oriented for FFS but not for CAP patients and profit-oriented at the same time, in both sequences. Another physician (#3) was benefit-oriented for FFS patients in S1 but not in S2.

### Classification result

One out of two physicians can be classified as a profit maximizer. Benefit maximizers occur but are rare. The remaining physicians show mixed motives; only a few of them are consistent in their tendencies. These classification results are in line with Hypothesis 4.

## Results

Our analysis of the experimental data relied on non-parametric methods. Such methods are particularly suitable when the sample size is small, and data are not assumed to result from prescribed models (the normal distribution model, for example). The non-parametric analysis is often the preferred method for comparing different treatments in an experimental study since it relies on ordinal data that are often not very sensitive to numerical data changes.

**Fig. 3:**
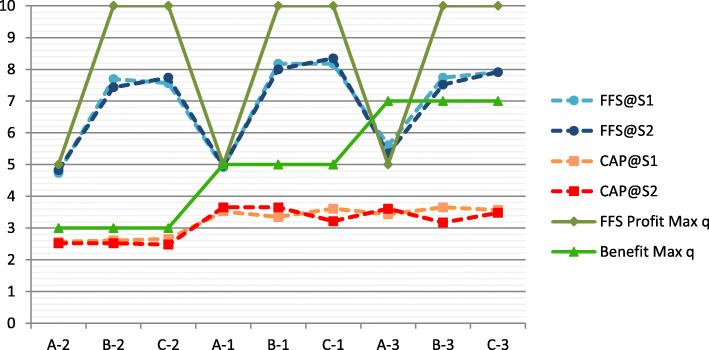
Average number of medical services per individual patient (characterized by illness [A – C] and patient type [1–3])

Table 3 provides an overview of the summary statistics. Figure 3 graphs the average number of medical services provided by the experimental physicians to each patient (characterized by the combination of illness and treatment preference) in both sequences (S1 and S2) and payment systems (FFS and CAP). It also indicates the profit-maximizing quantity and the right amount of medical care for each patient.

**Table 3:** Summary statistics

	FFS@S1	FFS@S2	CAP@S1	CAP@S2
**Quantity of treatment**				
Average	6.95	6.90	3.22	3.14
Median	7.00	7.00	3.00	3.00
SD	2.25	2.35	1.95	1.95
**Profit (ECU)**				
Average	9.08	8.97	10.59	8.23
SD	2.63	2.84	1.36	1.25
% Max profit^a^	50.2	48.3	13.5	15.5
**Patient benefit (ECU)**				
Average	8.04	7.95	6.54	6.39
SD	1.93	2.07	3.99	4.04
% Max patient benefit^b^	26.6	24.2	44.4	42.5

^a^Percent of individual treatment decisions coinciding with profit-maximizing quantity

^b^Percent of individual treatment decisions resulting in maximum patient benefit

In this section, we analyze the over-or under-provision of services to patients under FFS and CAP in both sequences (Section 3.1), the customization of care (Section 3.2), the effect of a CAP payment reduction (Section 3.3), physician profit (Section 3.4), and patient benefit (Section 3.5). Our non-parametric analyses were based on 23 independent observations. All tests were two-sided Wilcoxon signed-ranks tests (WTs).

### Over- or under-provision of medical services

Figure 3 above shows customization in response to the heterogeneity in the payment system, but it also suggests ready-to-wear treatment for patients with illness B or C under FFS (whose treatment provides maximum physician profit with the maximum quantity) and type 1 and 3 CAP patients. Under CAP, a level of treatment for type 2 patients similar to the one observed for types 1 and 3 would be higher than the treatment that maximizes the patient’s benefit.

We observed that for seven of the nine patients under FFS, the average treatment level in both sequences exceeded the corresponding correct amount of medical care. FFS patients were over-served when the profit-maximizing quantity exceeds the patient’s optimal quantity of treatment.

We also observed that, in each sequence, the average level of treatment provided to the six FFS patients with a profit-maximizing quantity of 10 was roughly the same (around 8), irrespective of the patient’s illness and preference for medical attention. This implies that the extent of over-provision to these patients is higher, the lower the patient’s need for medical attention. For instance, FFS patients B-3 and C-3, who needed extensive medical attention, received just a little more medical service than optimal. In contrast, FFS patients B-2 and C-2, in need of limited medical attention, were considerably over-served.

Two FFS patients in both sequences, characterized by the attributes A-1 and A-3, received fewer medical services than optimal. While the FFS patient labeled A-1 was only marginally under-served (the correct amount of medical services coincides with the profit-maximizing quantity), the FFS patient labeled A-3 received significantly fewer services than optimal (the optimal amount of medical care for this FFS patient exceeds the profit-maximizing quantity).

CAP patients were under-served in all cases. The average number of medical services provided to the nine CAP patients in both sequences fell below the optimal amount of care for the respective patients. In each sequence, the average number of medical services provided to type 1 and 3 CAP patients was roughly the same, irrespective of the patient’s need for medical attention. The degree of under-provision depended on the patient type. Type 3 CAP patients, in need of extensive medical attention, were considerably under-served. In contrast, type 2 CAP patients in need of minor medical attention were under-served to a considerably lower degree.

To gain statistical evidence regarding the under- or over-provision of medical attention to FFS and CAP patients, we examined the individual physicians’ average treatment decisions. Additional file 1: Table C.1 lists, for each physician, the average treatment quantity, along with the mean deviation from the correct amount of medical services over all the decisions regarding patients of the same payment system (FFS or CAP) and sequence (S1 or S2). It is obvious that in both sequences of the experiment more services than optimal were given to FFS patients: in each sequence, the mean deviation from the correct number of medical services for FFS patients is positive for 21 of the 23 physicians, while one is negative and one is zero. This implies that under FFS, physicians tended to significantly over-provide (S1, S2: *p* < 0.01; WTs). Analysis of the mean deviation for CAP patients shows that CAP patients received significantly fewer services than optimal in each sequence. The mean deviation is negative for 21 (22) physicians in S1 (S2), implying that under CAP, physicians tended to significantly under-provide (S1, S2: *p* < 0.01; WTs).

#### Result 1

In a heterogeneous payment environment, physicians over-served patients whose treatments are paid under FFS and under-served those whose treatments are paid under CAP. Over- and under-provision depended on the patient type and thus on the patient’s need for medical attention. Under FFS, over-provision was higher, the lower the need for medical attention. Under CAP, under-provision increased as the need for medical attention increased. The results confirm Hypothesis 1.

### Customization of care?

Figure 3 shows that, in both sequences, each type of FFS patient received considerably more medical attention than the CAP counterpart with the same illness. On average, physicians provided 6.95 (6.90) units of medical services to FFS patients and 3.22 (3.14) units to CAP patients in S1 (S2). Thus, they provided, on average, over twice as many medical services to FFS patients than to CAP patients in both sequences (S1, S2: *p* < 0.01; WTs based on Additional file 1: Table C.1).

#### Result 2

In each sequence, physicians provided more medical services to FFS than to CAP patients. Our findings thus refute the notion that physicians develop a one-for-all approach to providing medical care. Physicians did customize care according to the payment system in a heterogeneous payment environment, confirming Hypothesis 2.

### CAP payment reduction

Our study’s major concern was to analyze the potential impact of a lump-sum payment reduction on physician provision behaviour. We investigated whether it leads to a quantity reduction to CAP patients and/or spillover concerning the treatment of FFS patients. Note that the patients’ optimal amount of medical care and profit-maximizing quantity remained unaffected by the CAP reduction.

Looking at Fig. 3 and comparing the average quantity for patients with the same payment system, illness, and treatment preferences across sequences, we find that physician treatment behaviour toward FFS and CAP patients remained practically unaffected by the lump-sum payment reduction in S2. Comparing the average quantity provided by each physician to patients of the same payment system across sequences substantiates this observation. We do not find a significant difference in treatment behaviour across sequences for either FFS or CAP patients (WTs).

When we analyze individual treatment decisions for matching pairs of patients (illness and treatment preferences) in the same payment system across sequences, the comparison of individual treatment decisions shows no significant difference in treatment behaviour toward FFS or CAP patients across sequences (WTs).

#### Result 3

In contrast to Hypothesis 3**,** physicians’ provision behaviour remained virtually unaffected by an ex-ante payment reduction under capitation. Physicians did not alter their treatment behaviour toward FFS and CAP patients to recoup lost income. We observed neither a decline in treatment quantities regarding CAP patients nor a spillover effect in the FFS payment system.

### Physician profit

In the experiment, physicians earned an average of 331.81 ECU (median 340.50 ECU; ranging from 257.10 ECU to 373.80 ECU), implying that the average physician profit amounts to 88.8% of the maximum achievable profit of 373.80 ECU. One single participant, physician #7, realized the maximum achievable profit. Five out of the 23 physicians (#2, #21, #16, #4 and #7; 21.7% of all physicians) brought in 95% or more of the maximum achievable profit.

Table 3 shows that physicians earned, on average, 9.08 ECU (8.97 ECU) per FFS patient and 10.59 ECU (8.23 ECU) per CAP patient in S1 (S2). Under FFS, the observed averages are 8.9% (10%) lower than the average maximum attainable profit of 9.97 ECU per patient (remember that a patient’s illness impacts the FFS profit function); under CAP, the observed averages are 11.8% (14.3%) lower than the maximum profit of 12.00 ECU (9.60 ECU) per patient (profit under CAP remains unaffected by illness; lump-sum payments differ across sequences) in S1 (S2). Physicians gave up relatively more money relative to the maximum attainable profit under CAP than under FFS. For each physician, looking at the relative deviation of their profit from the maximum achievable profit, averaged over all patients of the same payment system, we find the relative deviation under CAP significantly larger than under FFS (S1, S2: *p* < 0.01; WTs).

To investigate whether the payment system affected the decision to choose the profit-maximizing treatment quantity, see Table 3. Under FFS, approximately half of each sequence’s individual treatment decisions coincided with the respective profit-maximizing quantity. In contrast, fewer than one in seven (six) individual treatment decisions resulted in maximum physician profit under CAP in S1 (S2). WTs based on the percentage of profit-maximizing decisions by each physician show that the relative share of profit-maximizing treatment decisions in each sequence is significantly larger under FFS than under CAP (S1, S2: *p* < 0.01).

#### Result 4

In the experiment, nearly one-quarter of the physicians earned 95% or more of the maximum attainable profit, indicating that they likely worked hard to maximize their overall profit. The payment system used to pay for a patient’s treatment influenced the physicians’ choice of profit-maximizing treatment quantities: while about half of all decisions under FFS maximized payoff, maximization occurred only occasionally under CAP (in both sequences).

### Patient benefit

Table 3 shows an average patient benefit of 8.04 ECU (7.95 ECU) for FFS patients and 6.54 ECU (6.39 ECU) for CAP patients in S1 (S2). The average benefits observed were thus somewhat lower for CAP patients than for FFS patients. Had physicians always acted in the patients’ best interest by providing the correct amount of medical care in every instance, both FFS and CAP patients could have received a maximum average benefit of 9.82 ECU (the experimental design distinguishes between three patient types with different benefit functions). In short, patients did not regularly receive optimal care (see Section 3.1).

To substantiate this finding, consider, for each physician, the average benefit received by FFS and CAP patients in each of the two sequences. WTs, based on comparisons of these averages with the maximum average patient benefit, show that patients under each payment system in each sequence received a significantly smaller benefit from medical treatment than under optimal treatment (FFS@S1, FFS@2, CAP@1, CAP@2: *p* < 0.01).

Table 3 lists the physicians’ relative shares of individual treatment decisions that resulted in optimal patient benefit. In each sequence, we find that roughly one in four individual treatment decisions (S1: 26.6%; S2: 24.2%) for FFS patients resulted in optimal patient benefit. However, 44.4% in S1 (42.5% in S2) of CAP patients’ individual treatment decisions resulted in optimal treatment. Comparing the relative share of optimal treatment decisions across payment systems for each physician, we find, for each sequence, the physicians’ relative share of optimal treatment decisions to be significantly larger for CAP than for FFS patients (S1, S2: *p* < 0.01; WTs).

To further assess the impact of the payment system on patient benefit, we look at the relative patient benefit loss, which is defined as the patient benefit loss (the difference between the maximum attainable patient benefit and the actual observed patient benefit) relative to the maximum achievable benefit. Analyzing the relative patient benefit loss averaged over all individual treatment decisions under the same payment system and sequence, we find in each sequence that FFS patients fared considerably better than CAP patients, despite the higher proportion of optimal treatment decisions for CAP than for FFS patients (Section 3.1).

Table 4 reports the relative patient benefit loss averaged over patients of the same illness but distinguished by type and the combination of payment system and sequence. It shows that in each sequence, type 1 FFS patients (in need of intermediate medical attention) and type 3 FFS patients (in need of extensive medical attention) did considerably better than their CAP counterparts (both, S1, S2: *p* < 0.01; WTs based on averages per physician). On the contrary, type 2 CAP patients (in need of minor medical attention) fared slightly better in S1 (*p* = 0.088) or roughly the same in S2 (*p* = 0.408) as their FFS counterparts.

**Table 4:** Patient benefit loss relative to the maximum possible

	FFS@S1	FFS@S2	CAP@S1	CAP@S2
Type 1	0.136	0.146	0.387	0.363
Type 2	0.209	0.209	0.162	0.211
Type 3	0.198	0.216	0.46	0.481
Average	0.181	0.19	0.336	0.352

To conclude, patients in need of intermediate or extensive medical attention fared better under FFS. Those in need of relatively little medical attention, though, if they experienced any effect of the payment system at all, suffered a lower benefit loss under CAP.

With regard to the effect of the lump-sum payment reduction under CAP, the lack of a significant behavioural volume response (Result 3) suggests that the patient benefit of FFS and CAP patients should remain largely unaffected. A comparison of the average benefit for patients of the same remuneration system across sequences supports this prediction. We found no significant change in the average patient benefit for FFS and CAP patients following the lump-sum payment reduction (see Table 3). For each physician, in terms of statistical evaluation, consider the average patient benefit for patients of the same payment system across sequences. The analysis showed no significant difference for either FFS or CAP patients (WTs).

We reported an insignificant change in the relative share of individual treatment decisions leading to optimal patient benefit for both FFS and CAP patients (see Table 3). Comparing, for each physician, the relative share of optimal treatment decisions for patients under the same payment system across sequences, we found no significant difference (WTs).

Comparing the average relative patient benefit loss for each patient type under FFS and CAP across sequences revealed minimal changes in response to the lump-sum payment reduction. Type 1 and 3 FFS patients fared slightly worse in S2 compared to S1 (see Table 4), while the average relative patient benefit loss for type 2 patients under FFS remained unaffected. Under CAP, type 1 patients fared slightly better, while type 2 and 3 patients fared slightly worse in S2 than S1. We did not find a significant difference when comparing the relative patient benefit loss for each physician over patients of the same type and payment system across sequences.

#### Result 5

Despite a higher proportion of CAP’s optimal treatment decisions than for FFS patients, patients ended up with a significantly lower benefit under CAP than under FFS. Benefit losses were significantly higher for CAP than for FFS patients in the case of patients in need of intermediate to major medical attention (two-thirds of our patients); this was not true for those in need of minor medical attention (one-third of our patients). This suggests that the overall benefit loss depends on the specific mix of patients with different needs. The CAP lump-sum payment reduction had no effect on treatment decisions, either for patients under CAP or under FFS. This is important news regarding opportunities for increasing taxpayer benefits by reducing costs under CAP.

## Discussion

### Findings and added value of our research

Our study adds to the existing experimental research by HSW (2011) on physician treatment behaviour [10] by incorporating a heterogeneous payment system, which is a prominent characteristic of markets for physician services in a number of countries (e.g., the United States). We did not directly compare pure FFS and pure CAP environments but instead focussed on physician behaviour in a heterogeneous practice environment where physicians faced comparable patients under FFS and CAP.

Our findings demonstrate the robustness of the results obtained by HSW (2011), more specifically, the tendency to over-serve FFS patients and under-serve CAP patients. We also investigated the effect of a CAP lump-sum payment reduction in a heterogeneous environment and found neither a direct effect on the treatment of CAP patients nor a spillover effect on FFS patients’ treatment.

Thus, our study complements the existing empirical and theoretical literature on the effects of financial incentives on physician behaviour. Our experimental study involved basic economic research and did not assess actual physician behaviour in the real world. Although the findings cannot be directly applied to real-world issues, they might provide valuable insights to those attempting to design new institutions. Laboratory experiments allow us to test the functioning of incentive schemes that do not yet exist in real life. They enable much tighter control than empirical studies or field experiments. Laboratory experiments are thus considered a useful complement to theoretical and empirical studies as well as field experiments.

If health care policy is to be designed effectively, there must be an in-depth consideration of the effects of payment system heterogeneity on physician provision behaviour. Potential system-wide effects of fee regulation, targeting the reimbursement of treatment for one subgroup of patients, should be examined as well.

In our experimental environment, we found that physicians customized care in response to the heterogeneous payment system. A patient’s medical treatment was affected by the payment system used to compensate the attending physician. In the experiment, an FFS patient received considerably more medical care than the corresponding CAP patient with the same illness and treatment preference. We also observed physicians over-serve FFS patients and under-serve CAP patients. Over-provision and under-provision depended on the patient’s need for medical attention. Under FFS (CAP), over-provision (under-provision) decreased (increased) as the patient’s need for medical attention increased. Patients in need of significant medical attention appeared to fare considerably better under FFS than under CAP.

In our experimental design, we assumed the patients were passive. If we removed this assumption, we would expect reputational effects to come into play [11]. Perceived over- or under-providers could thus expect patients to be more likely to reject treatment recommendations, which in turn could potentially limit such physician behaviour.

### Limitations

As for any lab experiment, the external validity of our findings remains an issue. Galizzi & Wiesen [26] mention three limitations: (1) participation bias (subjects that choose to participate in an experiment might be different from those who choose not to participate), (2) environment, context, and frame of the tasks in the lab might differ from decisions in real life, and (3) medical students may not be representative of the medical population. For the latter, Brosig-Koch et al. [18] and Reif et al. [17], for example, find no consistent difference in how physicians, medical and non-medical students respond to financial incentives in the field or in lab experiments. While the first limitation is a general issue with experimental economics, the second one can be tackled by redesigning tasks and contexts in additional experiments. Brosig-Koch et al. [27], for example, introduce competition into the experimental design of Brosig-Koch et al. [18, 21] and find that it can reduce over-provision (under FFS) as well as under-provision (under CAP).

In our experiment, the small number of participants might be an issue: it might have impaired the power of our statistical Wilcoxon tests. We investigate this issue using the G*Power software in the context of a post-hoc power analysis.^1^ The required significance level of our two-sided tests was set at 0.01. Therefore, when the null hypothesis (no effect) was rejected, even with only 23 observations, those tests’ power was close to 1 as expected and confirmed with the G*Power software. However, there was a difficulty in those situations, where the null hypothesis was accepted since the sample size might be too small to detect an effect. The power of the WTs was minimal. We recognized that it might be challenging to obtain the proper (huge) number to achieve a relevant power to the appropriate statistical tests in lab experiments. Furthermore, as seen in our results for the differential in the quantity of treatment with the reduction of CAP payment, the effect is likely to be very small if significant.

## Conclusion

Our results suggest that, in our experimental model, fee regulation could be used to some extent as a means of controlling physician spending since we did not identify a behavioural response to the CAP payment cut. Physicians did not recoup lost income by altering treatment behaviour toward CAP and/or FFS patients. The patient benefit under FFS and CAP thus appeared to remain unaffected by fee regulation. This finding relates to Brosig-Koch et al. [12] ‘s observation that, within a CAP payment system where performance pay appears to enhance the quality of care, the size of the bonus does not matter.

In line with the observations of HSW (2011) and Brosig-Koch et al. [18], our findings indicate that neither FFS nor CAP encourages physicians to provide optimal care from a patient’s perspective. This observation supports a move away from pure payment systems toward hybrid compensation schemes that blend the high- and low-intensity incentives embedded in FFS and CAP. The validity of results concerning the real world remains an issue with lab experiments. Running costly field experiments [12] and exploiting natural experiments are likely to improve this validity. Nevertheless, the field of experimental economics is a useful tool for further investigation that considers the welfare of all those involved in the health system and explores, for example, mixed CAP/FFS payment systems designed to reduce the under- and over-provision of specific health services. Another interesting avenue of research is the study of payment incentives to develop efficient teams of care providers.

## Supplementary Information

**Additional file 1: **Instructions. **Table B1.** Benefit functions for patient types 1, 2 and 3 (in ECU). **Table B2.** Physician profit. **Table C.1.** Quantity q and mean deviation from the optimal quantity q*, by physician, sorted with respect to overall profit (ascending).

## Data Availability

Fully anonymized datasets are available from the corresponding author upon request.

## Abbreviations

CAP: Capitation
CIRANO: Centre interuniversitaire de recherche en analyse des organisations
CAN$: Canadian dollar
ECU: Experimental currency unit
FFS: Fee-for-service
HSW: Hennig-Schmidt, Selten and Wiesen (2011)
OECD: Organisation for Economic Cooperation and Development
S1: First sequence of 18 patients presented to participants
S2: Second sequence of 18 patients presented to participants
WT: Wilcoxon signed ranks test

## Footnotes

1 We are indebted to a referee, who was guiding us to this software. https://www.psychologie.hhu.de/arbeitsgruppen/allgemeine-psychologie-und-arbeitspsychologie/gpower.html.
