# Supplementary material for: Custom-made health-care: an experimental investigation
Source: Health Econ Rev. 2020 Dec 18;10:41. doi: 10.1186/s13561-020-00299-4 (PMC7749502; doi:10.1186/s13561-020-00299-4)
Supplement: Supplementary file 1 — Additional file 1: Instructions. Table B1. Benefit functions for patient types 1, 2 and 3 (in ECU). Table B2. Physician profit. Table C.1. Quantity q and mean deviation from the optimal quantity q*, by physician, sorted with respect to overall profit (ascending). [file 13561_2020_299_MOESM1_ESM.docx]

# Online Appendices

# Appendix A: Instructions

**Experiment instructions**

You are participating in an experiment in which you will make independent and anonymous decisions. Depending on these decisions you can earn money.

All amounts in the experiment are denoted in ECU (Experimental Currency Units). The ECU that you earn in the experiment will be converted into $ CAD with a factor 1 ECU = 0.02 $ CAD and paid to you in addition to a show-up fee of 5 $ CAD in cash at the end of the experiment.

**your decisions**

In the experiment you will be in the role of a physician making medical decisions for virtual patients. These decisions will impact your profit as a physician as well as the patient benefit.

You will be responsible for the medical treatment of 36 virtual patients and decide for each individual patient on the number of medical services that you want to provide to this patient. The treatment can consist of an amount between zero (including) and ten (including) units of medical services.

The virtual patients will be presented to you one patient after the other. Each patient suffers from one out of three potential illnesses and belongs to one out of three patient types. We shall specify neither the illnesses nor the patient types in more detail. You won’t know the illness or type of a patient; you will only see numbers associated with illness and type related to possible treatments for the specific patient.

**your remuneration**

Your treatment will be remunerated either based on a Fee-for-Service (FFS) or a CAPITATION system. The remuneration system will vary across patients; the presentation of each patient includes information on the respective remuneration system.

If you treat a patient, for whom your services are remunerated based on FFS, each unit of service that you provide will be paid separately. Your remuneration thus increases with the number of services. In addition, your remuneration depends on the patient’s illness.

If you treat a patient for whom your services are remunerated by CAPITATION, you will receive a fixed payment of 12 ECU for this patient. This lump-sum payment is independent of the number of services provided by you and of the patient’s illness. You receive this payment even if you decide to provide zero service.

**your costs and profit**

With your decision on the number of medical services that you want to provide to a patient you also determine your costs of treating this patient. The treatment costs increase with the number of services.

Your profit per patient is determined by your remuneration minus your treatment costs for this patient.

**Monetary patient benefit**

Your decision on the number of medical services that you want to provide to a patient also determines the benefit that this patient gets from your treatment. This benefit depends on the patient type and the number of services but not on the illness.

**your information**

In the experiment we shall confront you with decision situations as in the following two examples. The examples consider the FFS and the CAPITATION remuneration scheme, respectively. In the experiment we shall present you a sequence of 36 such decision situations.

In the first example you have to make a decision on the number of services for a patient under FFS. The table shows you for each potential number of services (between zero and ten) that you provide to this patient with her specific illness your respective remuneration, your treatment costs, your profit (remuneration minus costs), and the monetary patient benefit that depends on the patient type You will be asked to enter your decision on the number of services units in the box below the table. Please choose an integer number between zero and ten. To confirm your decision, please click on “OK”.

| **Example FFS** |  |  |  |  |
| --- | --- | --- | --- | --- |
| Number of Services | Remuneration | Costs | Your Profit | Patient Benefit |
| 0 | 0.00 | 0.00 | 0.00 | 0.00 |
| 1 | 1.70 | 0.10 | 1.60 | 0.75 |
| 2 | 3.40 | 0.40 | 3.00 | 1.50 |
| 3 | 5.10 | 0.90 | 4.20 | 2.00 |
| 4 | 5.80 | 1.60 | 4.20 | 7.00 |
| 5 | 10.50 | 2.50 | 8.00 | 10.00 |
| 6 | 11.00 | 3.60 | 7.40 | 9.50 |
| 7 | 12.10 | 4.90 | 7.20 | 9.00 |
| 8 | 13.50 | 6.40 | 7.10 | 8.50 |
| 9 | 14.90 | 8.10 | 6.80 | 8.00 |
| 10 | 16.60 | 10.00 | 6.60 | 7.50 |

Please enter the number of services that you want to provide to this patient:

OK

The second example requires a treatment decision for a patient under CAPITATION. Here again you are provided for each number of services units the corresponding information on remuneration, costs, profit and monetary patient benefit.

**Example CAPITATION**

| Number of services | Remuneration | Costs | Your profit | Patient benefit |
| --- | --- | --- | --- | --- |
| 0 | 12.00 | 0.00 | 12.00 | 0.00 |
| 1 | 12.00 | 0.10 | 11.90 | 1.00 |
| 2 | 12.00 | 0.40 | 11.60 | 1.50 |
| 3 | 12.00 | 0.90 | 11.10 | 10.00 |
| 4 | 12.00 | 1.60 | 10.40 | 9.50 |
| 5 | 12.00 | 2.50 | 9.50 | 9.00 |
| 6 | 12.00 | 3.60 | 8.40 | 8.50 |
| 7 | 12.00 | 4.90 | 7.10 | 8.00 |
| 8 | 12.00 | 6.40 | 5.60 | 7.50 |
| 9 | 12.00 | 8.10 | 3.90 | 7.00 |
| 10 | 12.00 | 10.00 | 2.00 | 6.50 |

Please enter the number of services that you want to provide to this patient:

OK

**payment**

At the end of the experiment your individual profit resulting from the treatment of all 36 patients will be summed up, converted into $ CAD [1 ECU = 0.04 $ CAD] and paid to you in cash.

Since there are no real patients participating in this experiment, we shall donate the sum of patient benefits to a charitable healthcare organization. In this way your treatment decisions create benefit to real patients.

At the beginning of the experiment, you may decide on the charitable healthcare organization to which you want to donate. You can choose among:

- canadian cancer society(www.cancer.ca)
- multiple sclerosis society of canada (www.mssociety.ca)
- parkinson society of canada (www.parkinson.ca)

The patient benefits resulting from your decisions will we added up for all patients, converted into $ CAD with the same conversion factor as your own profit and paid to the organization of your choice.

The total patient benefit that has been created by all participants having chosen the same charitable healthcare organization will be donated online to the respective organization. We will do the payment in front of you at the end of the session.

Please turn now to the computer with your participation number and click on “Start”. You will be requested to answer a number of questions related to the understanding of these instructions. If you should have remaining questions, we will come to your workplace and answer them individually. As soon as all participants will have correctly answered all questions, the experiment can start.

**Appendix B: Additional tables (experimental protocol)**

Table B1: Benefit functions for patient types 1, 2 and 3 (in ECU)

|  | Quantity of medical services | | | | | | | | | | |
| --- | --- | --- | --- | --- | --- | --- | --- | --- | --- | --- | --- |
| Patient type | 0 | 1 | 2 | 3 | 4 | 5 | 6 | 7 | 8 | 9 | 10 |
| Type 1 | 0.00 | 0.75 | 1.50 | 2.00 | 7.00 | 10.00* | 9.50 | 9.00 | 8.50 | 8.00 | 7.50 |
| Type 2 | 0.00 | 1.00 | 1.50 | 10.00* | 9.50 | 9.00 | 8.50 | 8.00 | 7.50 | 7.00 | 6.50 |
| Type 3 | 0.00 | 0.75 | 2.20 | 4.05 | 6.00 | 7.75 | 9.00 | 9.45* | 8.80 | 6.75 | 3.00 |

* Interior global optimum

| Table B2: Physician profit | | | | | | | | | | | | | |
| --- | --- | --- | --- | --- | --- | --- | --- | --- | --- | --- | --- | --- | --- |
|  | | | | | | | | | | | | | |
|  |  |  | Quantity of medical services | | | | | | | | | | |
| Rem. syst. | Seq. | Illness | 0 | 1 | 2 | 3 | 4 | 5 | 6 | 7 | 8 | 9 | 10 |
| FFS | S1,S2 | A | 0.00 | 1.60 | 3.00 | 4.20 | 4.20 | 8.00 | 7.40 | 7.20 | 7.10 | 6.80 | 6.60 |
| FFS | S1,S2 | B | 0.00 | 1.70 | 3.20 | 4.50 | 5.60 | 6.50 | 7.20 | 7.70 | 8.00 | 8.10 | 8.30 |
| FFS | S1,S2 | C | 0.00 | 1.90 | 3.60 | 5.10 | 6.40 | 5.70 | 11.40 | 12.00 | 12.50 | 13.20 | 13.60 |
| CAP | S1 | A,B,C | 12.00 | 11.90 | 11.60 | 11.10 | 10.40 | 9.50 | 8.40 | 7.10 | 5.60 | 3.90 | 2.00 |
| CAP | S2 | A,B,C | 9.60 | 9.50 | 9.20 | 8.70 | 8.00 | 7.10 | 6.00 | 4.70 | 3.20 | 1.50 | -0.40 |

**Appendix C: Additional data tables**

Table C.1: Quantity q and mean deviation from the optimal quantity q*, by physician, sorted with respect to overall profit (ascending)

|  |  |  | | | | | | | | | | | | | | |
| --- | --- | --- | --- | --- | --- | --- | --- | --- | --- | --- | --- | --- | --- | --- | --- | --- |
|  |  | FFS@S1 | | | | | | |  | CAP@S1 | | | | | | |
|  |  | Quantity q | | |  | Deviation from q* | | |  | Quantity q | | |  | Deviation from q* | | |
| Physician # |  | Mean | Median | SD |  | Mean | Median | SD |  | Mean | Median | SD |  | Mean | Median | SD |
| 3 |  | 3.67 | 5.00 | 2.6 |  | -1.33 | -1.00 | 2.69 |  | 2.67 | 3.00 | 1.00 |  | -2.33 | -1.00 | 2.24 |
| 11 |  | 5.00 | 5.50 | 1.73 |  | 0.00 | 0.00 | 0.00 |  | 5.56 | 5.00 | 2.40 |  | 0.56 | 0.00 | 1.67 |
| 12 |  | 6.22 | 7.00 | 1.64 |  | 1.22 | 0.00 | 1.92 |  | 5.00 | 5.00 | 1.73 |  | 0.00 | 0.00 | 0.00 |
| 10 |  | 5.56 | 5.00 | 1.42 |  | 0.56 | 0.00 | 0.73 |  | 4.67 | 5.00 | 1.32 |  | -0.33 | 0.00 | 0.50 |
| 14 |  | 5.67 | 6.00 | 0.87 |  | 0.67 | 0.00 | 1.50 |  | 4.56 | 5.00 | 1.59 |  | -0.44 | 0.00 | 0.73 |
| 23 |  | 5.78 | 6.00 | 0.97 |  | 0.78 | 1.00 | 1.56 |  | 4.44 | 5.00 | 1.13 |  | -0.56 | 0.00 | 0.88 |
| 1 |  | 6.56 | 7.00 | 1.24 |  | 1.56 | 2.00 | 2.24 |  | 4.56 | 5.00 | 1.24 |  | -0.44 | 0.00 | 0.73 |
| 9 |  | 5.56 | 5.50 | 1.01 |  | 0.56 | 0.00 | 1.42 |  | 3.89 | 4.00 | 0.78 |  | -1.11 | 0.00 | 1.45 |
| 13 |  | 7.11 | 6.00 | 1.62 |  | 2.11 | 1.00 | 2.57 |  | 4.67 | 5.00 | 1.32 |  | -0.33 | 0.00 | 0.50 |
| 19 |  | 7.44 | 8.00 | 1.94 |  | 2.44 | 2.00 | 2.70 |  | 4.33 | 4.00 | 1.22 |  | -0.67 | -1.00 | 0.71 |
| 5 |  | 7.44 | 8.00 | 1.94 |  | 2.44 | 2.00 | 2.79 |  | 3.78 | 4.00 | 0.67 |  | -1.22 | -1.00 | 1.20 |
| 6 |  | 7.89 | 9.50 | 2.26 |  | 2.89 | 3.00 | 2.76 |  | 1.00 | 1.00 | 0.00 |  | -4.00 | -4.00 | 1.73 |
| 15 |  | 7.00 | 8.00 | 1.73 |  | 2.00 | 2.00 | 2.45 |  | 3.67 | 4.00 | 0.71 |  | -1.33 | -1.00 | 1.41 |
| 17 |  | 7.11 | 7.50 | 1.54 |  | 2.11 | 2.00 | 1.83 |  | 3.78 | 4.00 | 0.67 |  | -1.22 | -1.00 | 1.20 |
| 20 |  | 7.22 | 8.00 | 2.49 |  | 2.22 | 2.00 | 2.49 |  | 2.78 | 3.00 | 2.28 |  | -2.22 | -1.00 | 2.86 |
| 22 |  | 7.89 | 6.00 | 2.32 |  | 2.89 | 2.00 | 3.22 |  | 3.44 | 3.00 | 1.01 |  | -1.56 | -1.00 | 1.74 |
| 8 |  | 8.11 | 6.00 | 2.37 |  | 3.11 | 2.00 | 3.10 |  | 2.33 | 3.00 | 1.22 |  | -2.67 | -3.00 | 2.12 |
| 18 |  | 8.00 | 8.00 | 2.45 |  | 3.00 | 3.00 | 3.24 |  | 1.56 | 0.00 | 1.94 |  | -3.44 | -5.00 | 3.36 |
| 2 |  | 8.00 | 5.00 | 2.35 |  | 3.00 | 2.00 | 3.16 |  | 3.00 | 3.00 | 1.00 |  | -2.00 | -2.00 | 2.00 |
| 21 |  | 7.67 | 8.00 | 2.24 |  | 2.67 | 2.00 | 3.24 |  | 1.89 | 1.00 | 1.90 |  | -3.11 | -2.00 | 3.18 |
| 16 |  | 8.33 | 8.00 | 2.5 |  | 3.33 | 3.00 | 3.04 |  | 1.89 | 2.00 | 1.54 |  | -3.11 | -3.00 | 2.98 |
| 4 |  | 8.22 | 8.5 | 2.44 |  | 3.22 | 3.00 | 2.91 |  | 0.56 | 1.00 | 0.53 |  | -4.44 | -5.00 | 1.94 |
| 7 |  | 8.33 | 10.00 | 2.5 |  | 3.33 | 3.00 | 3.04 |  | 0.00 | 0.00 | 0.00 |  | -5.00 | -5.00 | 1.73 |
|  |  |  |  |  |  |  |  |  |  |  |  |  |  |  |  |  |
|  |  |  |  |  |  |  |  |  |  |  |  |  |  |  |  |  |

Table C.1: Quantity q and mean deviation from the optimal quantity q*, by physician, sorted with respect to overall profit (ascending) (cont.)

|  |  |  | | | | | | | | | | | | | | |
| --- | --- | --- | --- | --- | --- | --- | --- | --- | --- | --- | --- | --- | --- | --- | --- | --- |
|  |  |  | | | | | | | | | | | | | | |
|  |  | FFS@S2 | | | | | | |  | CAP@S2 | | | | | | |
|  |  | Quantity q | | |  | Deviation from q* | | |  | Quantity q | | |  | Deviation from q* | | |
| Physician # |  | Mean | Median | SD |  | Mean | Median | SD |  | Mean | Median | SD |  | Mean | Median | SD |
| 3 |  | 2.11 | 2.00 | 0.78 |  | -2.89 | -2.00 | 2.26 |  | 1.22 | 1.00 | 0.67 |  | -3.78 | -3.00 | 1.56 |
| 11 |  | 5.00 | 5.00 | 1.73 |  | 0.00 | 0.00 | 0.00 |  | 5.00 | 5.00 | 1.73 |  | 0.00 | 0.00 | 0.00 |
| 12 |  | 5.56 | 6.00 | 1.67 |  | 0.56 | 0.00 | 1.51 |  | 4.67 | 5.00 | 1.41 |  | -0.33 | 0.00 | 0.71 |
| 10 |  | 5.89 | 6.00 | 1.27 |  | 0.89 | 1.00 | 1.05 |  | 4.67 | 5.00 | 1.32 |  | -0.33 | 0.00 | 0.50 |
| 14 |  | 6.00 | 7.00 | 1.58 |  | 1.00 | 0.00 | 1.58 |  | 4.78 | 5.00 | 1.48 |  | -0.22 | 0.00 | 0.44 |
| 23 |  | 5.89 | 6.00 | 1.05 |  | 0.89 | 1.00 | 1.05 |  | 4.67 | 5.00 | 1.32 |  | -0.33 | 0.00 | 0.50 |
| 1 |  | 6.89 | 7.00 | 1.36 |  | 1.89 | 2.00 | 1.96 |  | 4.44 | 5.00 | 1.13 |  | -0.56 | 0.00 | 0.88 |
| 9 |  | 5.44 | 6.00 | 1.13 |  | 0.44 | 0.00 | 1.51 |  | 3.67 | 4.00 | 0.50 |  | -1.33 | -1.00 | 1.32 |
| 13 |  | 7.89 | 8.00 | 1.62 |  | 2.89 | 3.00 | 2.37 |  | 3.44 | 1.00 | 2.35 |  | -2.67 | -2.00 | 2.55 |
| 19 |  | 7.33 | 8.00 | 1.87 |  | 2.33 | 2.00 | 2.74 |  | 4.00 | 4.00 | 0.87 |  | -1.00 | 0.00 | 1.32 |
| 5 |  | 7.22 | 8.00 | 1.72 |  | 2.22 | 2.00 | 2.54 |  | 4.11 | 4.00 | 1.54 |  | -0.89 | -1.00 | 1.96 |
| 6 |  | 6.33 | 6.00 | 2.40 |  | 1.33 | 1.00 | 3.28 |  | 2.33 | 1.00 | 2.50 |  | -2.67 | -4.00 | 2.78 |
| 15 |  | 7.00 | 8.00 | 1.58 |  | 2.00 | 2.00 | 2.24 |  | 3.33 | 4.00 | 1.00 |  | -1.67 | -1.00 | 2.00 |
| 17 |  | 7.33 | 8.00 | 1.87 |  | 2.33 | 2.00 | 2.45 |  | 3.67 | 4.00 | 0.50 |  | -1.33 | -1.00 | 1.32 |
| 20 |  | 8.00 | 10.00 | 2.45 |  | 3.00 | 3.00 | 3.24 |  | 2.89 | 3.00 | 1.90 |  | -2.11 | 0.00 | 2.85 |
| 22 |  | 8.11 | 9.00 | 2.20 |  | 3.11 | 2.00 | 2.98 |  | 4.00 | 4.00 | 0.87 |  | -1.00 | -1.00 | 1.12 |
| 8 |  | 8.11 | 10.00 | 2.42 |  | 3.11 | 3.00 | 3.14 |  | 3.56 | 3.00 | 1.13 |  | -1.44 | 0.00 | 2.19 |
| 18 |  | 7.33 | 7.00 | 2.24 |  | 2.33 | 2.00 | 3.39 |  | 2.67 | 3.00 | 2.18 |  | -2.33 | 0.00 | 3.12 |
| 2 |  | 8.00 | 9.00 | 2.35 |  | 3.00 | 2.00 | 3.16 |  | 2.89 | 3.00 | 0.60 |  | -2.11 | -2.00 | 1.96 |
| 21 |  | 8.22 | 10.00 | 2.44 |  | 3.22 | 3.00 | 3.07 |  | 2.11 | 2.00 | 1.54 |  | -2.89 | -3.00 | 2.42 |
| 16 |  | 8.33 | 10.00 | 2.50 |  | 3.33 | 3.00 | 3.04 |  | 1.22 | 0.00 | 1.64 |  | -3.78 | -5.00 | 3.27 |
| 4 |  | 8.33 | 10.00 | 2.50 |  | 3.33 | 3.00 | 3.04 |  | 0.11 | 0.00 | 0.33 |  | -4.89 | -5.00 | 1.76 |
| 7 |  | 8.33 | 10.00 | 2.50 |  | 3.33 | 3.00 | 3.04 |  | 0.00 | 0.00 | 0.00 |  | -5.00 | -5.00 | 1.73 |
|  |  |  |  |  |  |  |  |  |  |  |  |  |  |  |  |  |
